# Influence of working memory overload on emotional processing and recognition memory: An fNIRS study

**DOI:** 10.3758/s13421-025-01746-5

**Published:** 2025-06-18

**Authors:** Cristina Moya, Nieves Fuentes-Sánchez, Ma. Cruz Martínez-Sáez, Laura Ros, José M. Latorre

**Affiliations:** Department of Psychology, Faculty of Medicine, Universidad de Castilla-La Mancha, C/Almansa, 14, Albacete, Spain

**Keywords:** Working memory, Emotion, Recognition memory, Prefrontal cortex, FNIRS

## Abstract

The aim of this study was to investigate the impact of working memory overload on emotional processing and recognition memory. Firstly, to study emotional processing, subjective and fNIRS correlates were measured while inducing emotions using affective pictures presented for 6 s. A recognition memory task was then administered, in which participants were required to indicate whether each affective stimulus was new or had previously been used in the passive viewing task. A sample of 70 healthy volunteers (44 women) were divided into an experimental group in which working memory was overloaded during the emotion induction procedure, and a control group in which working memory was not overloaded. Regarding the effect of working memory overload on emotional processing, the results showed that the experimental group rated negative stimuli as less unpleasant. Additionally, this group presented higher fNIRS activations in the dorsolateral prefrontal cortex (DLPFC), particularly to high arousal stimuli. Meanwhile, the findings revealed better recognition for negative and high arousal stimuli in the experimental group. Overall, our findings provide further evidence on the modulation of emotional processing and recognition memory as a function of working memory overload, while highlighting the importance of the DLPFC in emotion processing and cognitive load management.

## Introduction

Executive functions are a set of skills that enable individuals to self-regulate, plan, monitor, and evaluate their performance in problem-solving (Zelazo & Frye, 1998) while also being involved in cognitive flexibility, inhibition of automatic responses, and the generation of goal-directed behaviours. Executive functions encompass subcomponents such as inhibitory control, cognitive flexibility, and working memory (Diamond, 2013), the last of which plays an essential role in diverse daily activities, including learning, problem-solving and decision-making (Spencer, 2020). According to Baddeley (2003), working memory functions include the ability to retain and manipulate information in the mind, to switch efficiently between maintenance and processing activities, to actively inhibit irrelevant content, and to orient and maintain attention on task goals. Thus, working memory allows us to keep relevant information in mind while carrying out complex cognitive activities. Its neurological foundations lie in the prefrontal cortex, a brain region known for its crucial role in executive functions, specifically in the dorsolateral prefrontal cortex (DLPFC; Balconi, 2013). In particular, neuroimaging studies have demonstrated that the prefrontal cortex is a key region in the temporary maintenance of intermediate results, planning, and temporal sequencing of tasks, result verification, and error connection (Jones & Graff-Radford, 2021), as well as in other functions related to working memory, such as the storage and retrieval of information required for solving mathematical activities (Hernández-Suárez et al., 2021). Together with other interconnected brain areas, including the temporal cortex, the posterior cortex and the inferotemporal lobe, it forms a network that underlies working memory (Gómez et al., 2018).

It is well known that working memory may be influenced by other psychological processes, such as emotional processing (Liu et al., 2018). This impact has been documented in several studies, albeit with contradictory findings. While some studies report that positive emotions benefit working memory (González-Garrido et al., 2015; Liu et al., 2018), others argue that negative emotions enhance this process (Xie & Zhang, 2016). Meanwhile, Zhang et al. (2017) found that both negative and positive emotions increased working memory capacity. In contrast to these findings, other studies have found no influence of emotional processing on working memory (Bannerman et al., 2012). Surprisingly, few works have examined the inverse relationship (i.e., how working memory influences emotional processing). In general, the scant literature in this regard has revealed that working memory overload decreases the emotional intensity reported (Grissmann et al., 2017; Kron et al., 2010). Specifically, Van Dillen and Koole (2007) conducted an experiment where participants viewed unpleasant stimuli, followed by a working memory task, and were then asked to rate the intensity of their feelings. The results showed that working memory overload decreased the intensity of feelings, suggesting that people can distract themselves from negative feelings through loading their working memory capacity. In the same vein, Kron et al. (2010) carried out two sets of experiments to investigate the relationship between emotion processing and working memory. The first experiment investigated how emotional arousal was influenced by a concurrent secondary task of backward counting. The results revealed a decrease in the emotional intensity of both unpleasant and pleasant emotions under cognitive load. Additionally, this attenuation of feeling was mediated by neither demand characteristics nor distraction of external stimuli. In the second experiment, cognition was overloaded by telling the participants to stay focused on feelings. As prior findings, the participants under cognitive load reported lower emotional intensity than those not subjected to cognitive load. The authors interpreted these results in relation to the mere resource hypothesis, which states that feelings require mental resources, such that introducing a task that creates cognitive load by using these resources will diminish the intensity of feelings (Kron et al., 2010). Meanwhile, Storbeck and Watson (2014) went a step further by distinguishing between verbal and spatial working memory, finding that completing a verbal working memory task led to more positive evaluations of the stimuli, whereas completing a spatial working memory task resulted in more negative evaluations of the stimuli. In the same line, studies by Gray (2004) and Storbeck (2012) found that positive affect enhances verbal working memory and negative affect enhances spatial working memory. Taken together, these findings suggest the existence of reciprocal associations between emotion and working memory. They also highlight the need to explore the effects of working memory in relation to emotion, distinguishing the type of working memory task (i.e., mathematical calculation update task).

The link between working memory and emotional processing is also supported by common neurophysiological correlates. Particularly, it has been demonstrated that the connection between subcortical areas, such as the limbic system and the basal ganglia with cortical regions, such as the ventromedial prefrontal cortex (vmPFC) or the DLPF, have important implications for both emotional processing and executive functions, as is the case of working memory (Fitzgerald et al., 2018; Phillips et al., 2003).

On the other hand, recognition memory, as a key component of declarative memory, depends on strategic processes that rely on working memory (Stebbins et al., 1999), and so it is also a process that can be influenced by this executive function. Specifically, the mediating role of working memory is related to the maintenance of information and the inhibition of irrelevant information, which influences attentional and mnestic processes (Oberauer, 2005). Although studies have examined the relationship between working memory and recognition memory (Alonso-Recio et al., 2014; Cotton & Ricket, 2021), research on the influence of working memory load on recognition memory is limited and suggests that such overload negatively impacts recognition memory (Castellà et al., 2020). At the neurophysiological level, common neural correlates underlie both processes, since the prefrontal cortex performs executive and attentional control functions that contribute to the appropriate processing of mnestic information (Tirapu-Ustárroz et al., 2012).

Given the importance of the relationship between working memory, emotional processing and recognition memory, research on this association is of great interest. In this respect, it has been observed that emotional stimuli, whether positive or negative, are better remembered than neutral ones (Laney et al., 2004). This effect is known as emotional enhancement of memory and has been evidenced in several studies (Kensinger & Corkin, 2004; Öhman et al., 2001). Interestingly, the advantage of the emotional stimuli on recognition might be affected when the working memory is overloaded, as argued by Miendlarzewska and collaborators (2013). Specifically, these authors revealed that the number of correctly recognized emotional stimuli—both positive and negative—decreased when the mnestic recognition test was presented while overloading working memory. This finding was not obtained, however, for neutral stimuli. In this line, prior research has investigated how the hedonic valence and arousal levels of the stimuli may influence recognition memory. On the one hand, Mather and Sutherland (2011) argued that affective stimuli with high arousal increase the priority of such stimuli in recognition memory, suggesting it is the most important factor. On the other, studies have found that hedonic valence mediates the effects between arousal and recognition memory (Mickley Steinmetz et al., 2010; Vuilleumier, 2005), with negatively valenced stimuli being those with the greatest recognition advantage (Alves et al., 2015; Moret-Tatay et al., 2014). Nevertheless, it is also worth mentioning that when studying the influence of emotion processing on recognition memory, its effects on response bias have been found to be more robust than on recognition accuracy (Dougal & Rotello, 2007; Rotello & Macmillan, 2007). In this respect, signal detection theory (SDT) provides a framework for understanding this distinction by separating memory sensitivity (recognition accuracy) from response bias (decision tendencies; Rotello & Macmillan, 2007). Prior research has shown that emotion does not consistently improve recognition accuracy and that many previously observed advantages of emotional stimuli in memory tasks may stem from shifts in response criteria rather than genuine enhancements in episodic recollection (Dougal & Rotello, 2007). In this sense, emotion creates the illusion of enhanced memory by increasing familiarity-based responses without necessarily improving the ability to accurately differentiate between previously studied and novel stimuli. Regarding the response criterion bias, as noted by Wylie et al. (2021), fatigue induced by a working memory task results in a more conservative criterion. Given the above, when interpreting the results of investigations that explore the effects of emotion on recognition memory, these considerations must be considered.

Besides the above significant findings, some methodological and theoretical caveats should be mentioned. Firstly, as mentioned, few studies have investigated the interplay between working memory, emotional processing, and recognition memory, with most of them having primarily focused on the effect of emotional processing on cognitive processes such as working memory or recognition memory, but not the other way around, thus making further studies necessary. In this line, previous works in the field have studied the effect of hedonic valence on emotional and cognitive processes (Grissman et al., 2017; Mirandola & Toffalini, 2018; Ozawa et al., 2014), but the interaction of this variable with arousal has been little investigated. Secondly, the prior literature has predominantly used subjective measures (e.g., self-ratings), but few works have considered other objective measures, such as near-infrared spectroscopy (fNIRS), an innovative technique to investigate brain responses that has numerous advantages over traditional method. In recent years, there has been growing use of fNIRS in research related to emotional induction and its neural correlates, with findings suggesting an increase in fNIRS activity in the prefrontal cortex in response to emotional stimuli, consistent with self-reported activation (Glotzbach et al., 2011; Hu et al., 2019; Struckmann et al., 2022; Yükselen et al., 2023). In this sense, this technique has shown good tolerance for artifacts produced by movement. Furthermore, during signal recording, it does not emit noise, an advantage when performing certain cognitive tasks. Likewise, fNIRS has shown a better spatial resolution than EEG and a better temporal resolution than fMRI (Pinti et al., 2020).

Considering these issues, the main objective of this study was to investigate the impact of working memory overload on emotional processing and recognition memory. To this end, self-reports (hedonic valence and arousal) and fNIRS were used to investigate subjective and brain correlates. Secondarily, we sought to investigate the effect of hedonic valence and arousal on emotional processing and information recognition. Furthermore, this study aimed to examine the role of DLPFC on working memory and emotional processing, as well as the interaction between both processes.

Regarding the impact of working memory overload on emotional processing, following previous studies (Dixon et al., 2017; Xie & Zhang, 2016; Zhang et al., 2017), we expected to find a decrease in perceived hedonic valence and arousal activation, as well as an increase in fNIRS activation in DLPFC when overloading working memory using a concurrent mathematical calculation update task while inducing emotions. Likewise, at the overall level, we hypothesized higher fNIRS activation in PFC during the viewing of pictures with high arousal—both positive and negative—(Glotzbach et al., 2011) as well as during the viewing of pictures with negative valence in comparison with positively valenced stimuli, as negative stimuli have been associated with greater prefrontal activity than positive and neutral stimuli (Hoshi et al., 2011; Ozawa et al., 2014). On the other hand, with regard to the effect of working memory overload on recognition memory, we posited a decrease in the emotional enhancement of memory during the viewing of emotional pictures that concurred with the working memory task. Additionally, at a general level, we expected better recognition of stimuli with high arousal (both positive and negative) in comparison with low arousal stimuli, as well as better recognition of negative stimuli (with high and low arousal) compared with positive stimuli.

## Method

### Participants

Sample size was calculated using G*Power 3.1 (Faul et al., 2009), considering an effect size (*f*) of 0.25, an alpha level of 0.05, a power value of 0.95, two groups (experimental, control), and four measurements (high arousal, low arousal, high valence, low valence), suggesting that at least 54 participants were needed. As an inclusion criterion, participants needed to be between 18 and 45 years old. Furthermore, participants with a diagnosis of psychiatric illness were excluded.

A total of 70 participants (44 women) between the ages of 18 and 42 years (*M*_age_ = 22.66 years, *SD*_age_ = 4.2) were finally recruited for the study. The sample consisted of volunteer students from the University of Castilla-La Mancha (Spain) as well other volunteers contacted through social networks. Of the initial sample, 16 participants were excluded from the fNIRS analyses due to technical problems during data acquisition. Therefore, statistical analyses were conducted with 54 participants for fNIRS, whereas 70 participants were included for the data analysis of affective ratings.

The study protocol was approved by the Social Research Ethics Committee of the University of Castilla-La Mancha (protocol number 06/2016). All the participants gave their written informed consent in accordance with the Declaration of Helsinki. Only undergraduate students were awarded course credits for their participation.

### Stimuli and design

Participants were randomly distributed to the experimental (*n* = 34) or the control group (*n* = 36). In the experimental group, participants were given a working memory task during the emotional passive viewing task, while control group participants were not.

For both groups, the experimental task was divided into two stages. Firstly, a passive viewing task was performed to elicit emotional responses. In this task, a total of 50 pictures (10 with positive valence and low arousal [P-L], 10 with positive valence and high arousal [P-H], 10 with negative valence and low arousal [N-L], 10 with negative valence and high arousal [N-H], and 10 neutral ones) were selected from the International Affective Picture System (IAPS; Lang et al., 2005).^1^ The normative mean values and standard deviations (*SD*) for each picture category are summarized in Table 1.^2^

**Table 1:** Normative means (*M*), standard deviations (*SDs*), and confidence intervals (CI) for each category in hedonic valence and arousal

	Hedonic valence			Arousal		
	95% CI			95% CI		
	*M* (*SD*)	Lower	Upper	*M* (*SD*)	Lower	Upper
**Neutral**	5.00 (0.67)	4.52	5.48	4.08 (0.66)	3.60	4.56
**P-H**	7.12 (0.58)	6.67	7.57	6.89 (0.74)	6.33	7.46
**P-L**	7.40 (0.63)	6.95	7.85	3.53 (0.27)	3.34	3.73
**N-H**	1.65 (0.34)	1.41	1.90	7.49 (0.37)	7.22	7.76
**N-L**	3.31 (0.64)	2.86	3.77	4.60 (0.84)	4.00	5.20

The emotional pictures were distributed into five blocks with ten pictures from each category. The presentation of the blocks was randomized with the exception of the neutral block, which was always the initial one, serving as a practice trial.

**Fig. 1:**
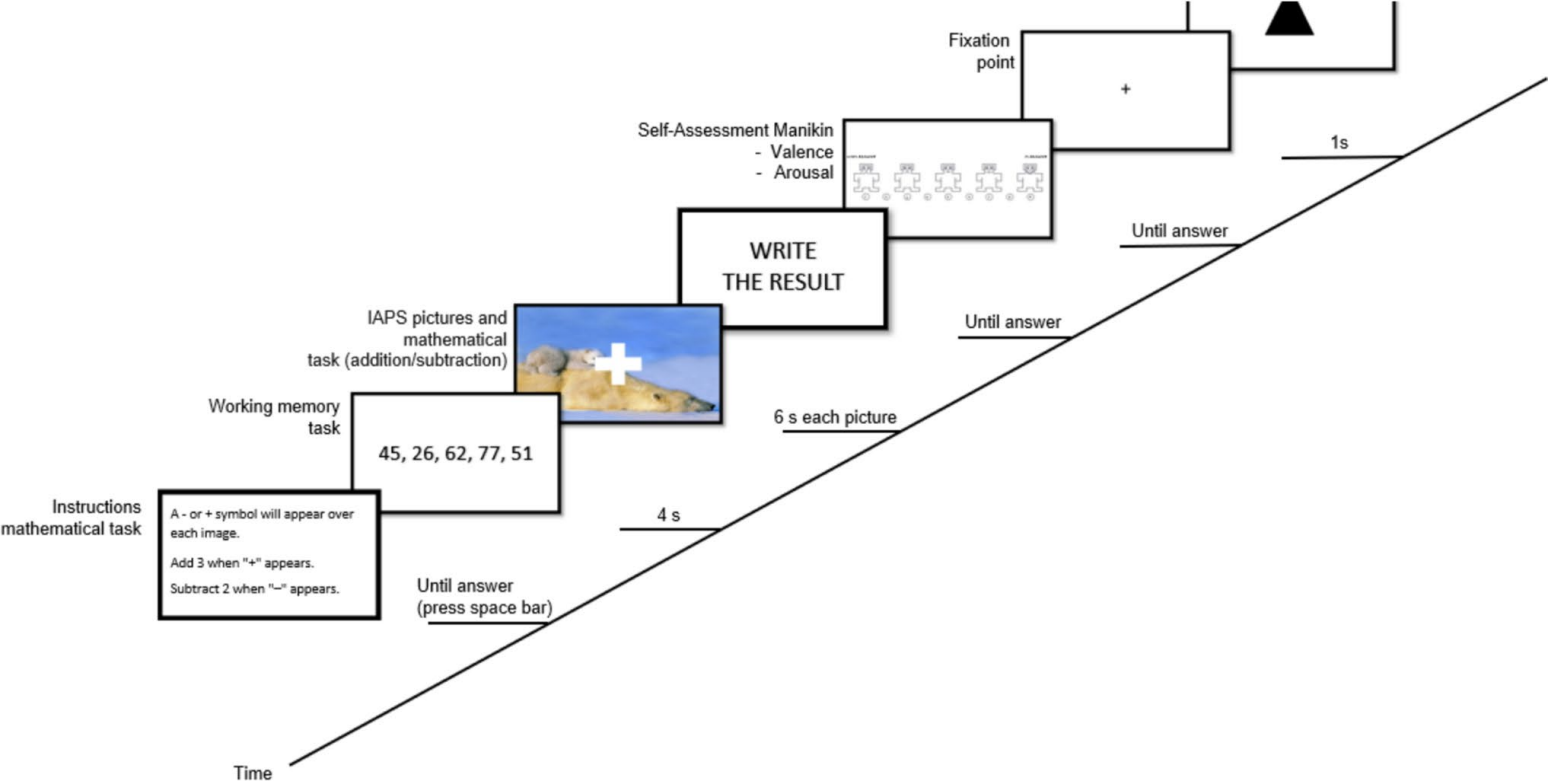
Trial structure of the passive viewing task for the experimental group

In the control group, each trial began with the 6-s presentation of the pictures. Subsequently, the participants rated hedonic valence (1 = *unpleasant* to 9 = *pleasant*) and arousal (1 = *relaxed* to 9 = *excited*) for all presented pictures, using the Self-Assessment Manikin (SAM; Bradley & Lang, 1994). Finally, each trial ended with a distraction task, which consisted of identifying a geometrical figure. In contrast to the control group, in the experimental group, the passive viewing tasks were simultaneously displayed with a concurrent working memory task. This task consisted of a mathematical calculation update task, which comprised a series of mental operations (additions and subtractions), which were performed based on a number given to the participant before starting each block. The operation to be performed depended on whether the image contained a plus or minus symbol, with all pictures containing one of these two mathematical symbols. Participants then rated the affective valence and arousal of each picture using the SAM and, finally, the trials ended with the distraction task (see Fig. 1).

**Fig. 2:**
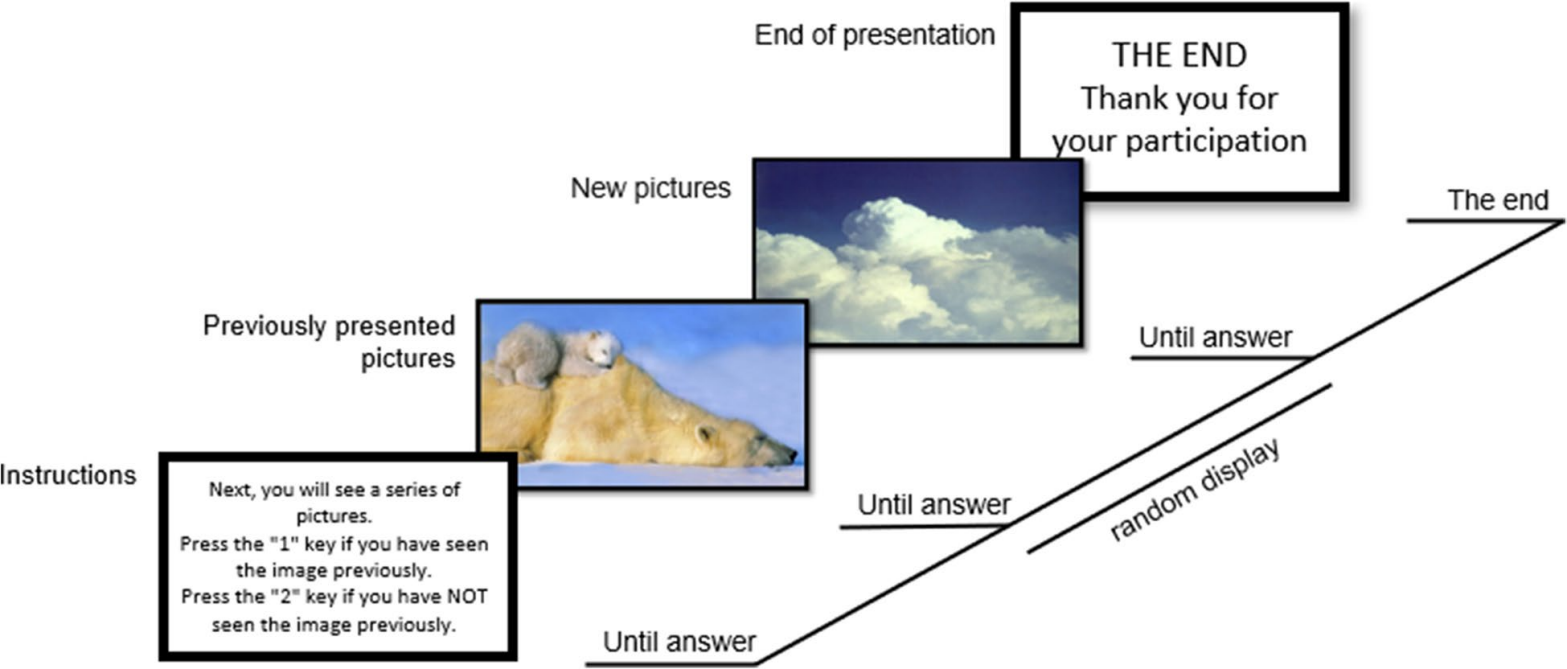
Trial structure for the recognition memory task

The second phase of the experimental task was a recognition memory task. This task consisted of the random presentation of 50 pictures (10 for each emotional category), of which 25 had previously been presented in the passive viewing task, and 25 were new.^3^ The normative mean values and standard deviations for the new 25 pictures used in the recognition memory task are summarized in Table 2. The 25 “old” pictures (those used in the previous passive viewing task) were randomly selected from the prior task, ensuring that there were five per emotional category, and the new 25 pictures were selected from the IAPS dataset controlling for similar valence and arousal levels. In the task, participants were asked to indicate whether each stimulus was new or had previously been used in the passive viewing task (see Fig. 2). If they answered correctly, this was considered a hit. This second stage completed the task.

**Table 2:** Normative means (*M*), standard deviations (*SDs*), and confidence intervals (CI) in hedonic valence and arousal for the new emotional pictures used in the recognition memory task, considering each category

	Hedonic valence			Arousal		
	95% CI			95% CI		
	M (SD)	Lower	Upper	M (SD)	Lower	Upper
Neutral	5.02 (0.18)	4.79	5.25	3.12 (0.96)	1.93	4.31
P-H	7.30 (0.37)	6.84	7.75	6.82 (0.48)	6.23	7.42
P-L	6.98 (0.71)	6.10	7.87	3.42 (0.30)	3.05	3.79
N-H	1.67 (0.24)	1.37	1.98	6.98 (0.12)	6.83	7.12
N-L	3.32 (0.39)	2.84	3.81	3.95 (0.21)	3.69	4.21

### Procedure

Each participant took part in one laboratory session, which lasted approximately 40 min. First, the participants read an overview of the task and completed a written consent form. Subsequently, they were given the instructions for the task and completed a brief survey to collect individual variables, including age and gender. The fNIRS device was then put in position and the calibration was performed. Finally, the experimental task started, lasting approximately 20 min. Once the task was over, the fNIRS was removed from the participant.

### fNIRS data acquisition and reduction

**Fig. 3:**
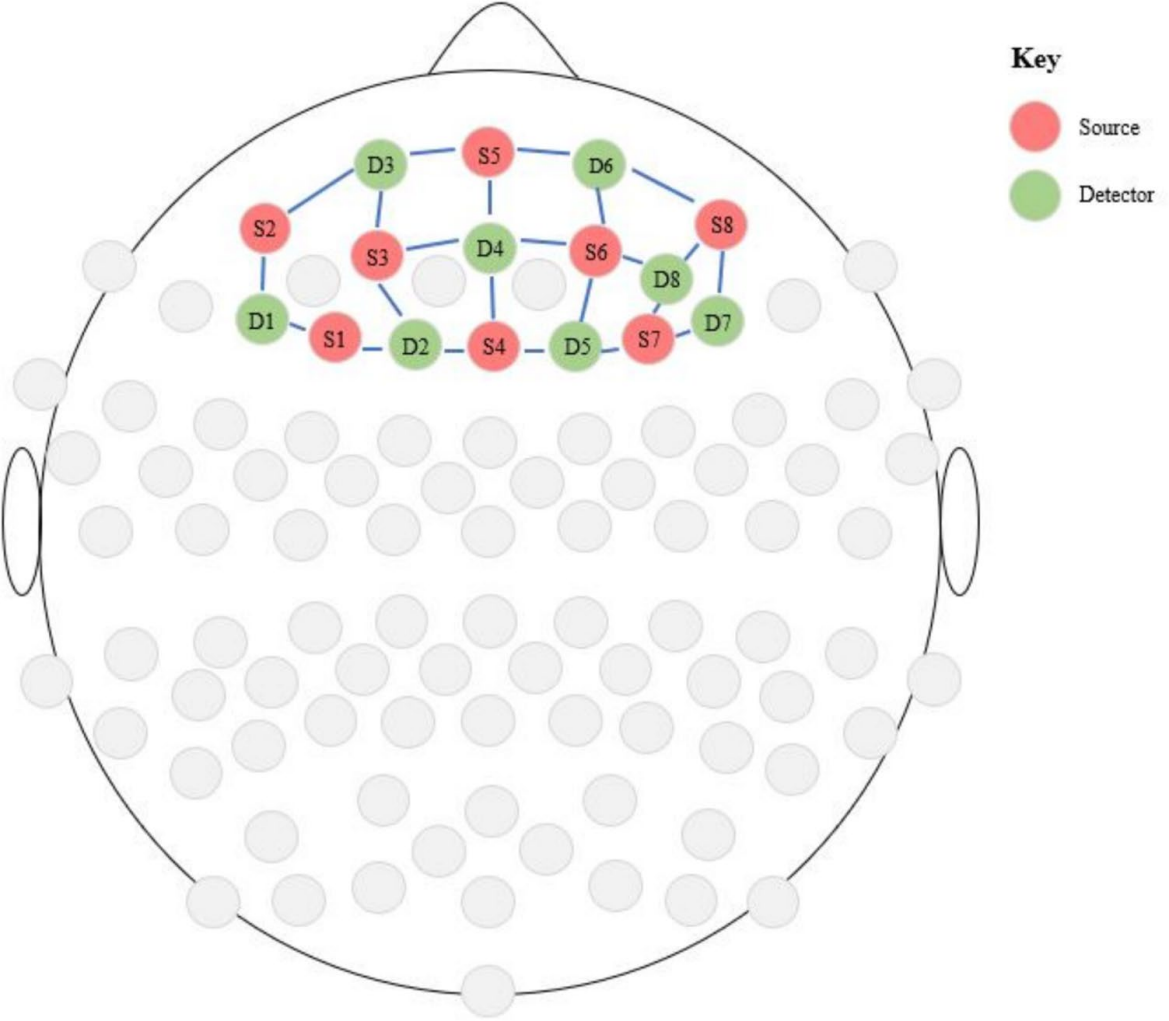
Sources and detectors positions for prefrontal measurements. (Color figure online)

Functional near-infrared spectroscopy (fNIRS) is an optical, noninvasive neuroimaging method that allows for the recording of neural activity by measuring changes in oxygenated (oxy-Hb) and deoxygenated hemoglobin (deoxy-R) through shining and collecting near-infrared light on the surface of an individual’s head (Hoshi, 2016). In this study, we used a continuous-wave NIRS device (NIRScoutX, New York), which employs 48 sources and 32 detectors. The NIRScout system was used with a 7.81-Hz sampling rate. Default Prefrontal 8x8 montage in international 10–10 coordinate space was used. In Fig. 3, the sources are indicated in red and the detectors in gray, while the purple lines correspond to channels.

To ascertain the placement of fNIRS optodes on the brain, we employed a technique that relies on sensitivity profiles derived from photon transport simulations carried out in two cranial discharges. The outcomes were consolidated into a tool called the “fNIRS Optodes’ Location Decider” (fOLD). This tool, using a predefined set of positions, optimizes the anatomical precision of the brain regions of interest according to the international 10–10 system (Jurcak et al., 2007). We selected the atlas that compiles Brodmann areas from the five parcellation atlases available in fOLD to define the regions of interest (ROIs; Rorden & Brett, 2000; Zimeo Morais et al., 2018). By aligning the sources and detectors of our 8 × 8 prefrontal montage with the Brodmann areas atlas, we identified and extracted the pertinent channels.

Finally, the brain regions included were the DLPFC (Brodmann Area 9: Channels 2, 5, 6, 10, 11, 13, 14, 14, 15, 77, 79, 83, and 84; Brodmann Area 46: Channels 4, 5, 6, 7, 8, 9, 14, 15, 77, 78, 79, 80, 81, 82, 83, and 84), orbitofrontal area (Brodmann Area 11; Channels 1, 2, 3, 4, 8, 78, and 81), dorsal anterior cingulate cortex (Brodmann Area 32; Channels 2, 5, 10, 11, and 77) and inferior prefrontal gyrus (Brodmann Area 47; Channels 8, 9, 81, and 82; see Fig. 3).

The raw optical data obtained from the fNIRS acquisitions were converted to optical density, and then into oxy- ([HbO]) and deoxyhemoglobin ([HbR]) relative concentration changes. For statistical analyses, the total of these two parameters was considered in the SPSS analysis.

### Data analyses

First, in order to investigate the effect of working memory overload on emotional processing, a repeated measure analysis of variance (ANOVA) with *valence* (positive, negative), and *arousal* (high, low) as the within-subject factor, and *group* (experimental, control) as the between-subject factor was calculated separately for each subjective rating (hedonic valence and arousal) and for fNIRS activations (separately for each area: 9, 46, 11, 32, and 47). Post hoc comparisons (Bonferroni test) were performed to explore plausible differences between conditions. Additionally, a *t* test comparison was conducted to explore the differences in self-ratings and fNIRS activations between the control and experimental group for the neutral block. Finally, a paired *t* test was performed in order to examine the fNIRS activation by Brodmann areas.

Second, with the aim of examining the effect of working memory overload on recognition memory, the number of hits in the recognition memory task was considered. Additionally, *d′* (*d* prime) and* c* (criterion) values were measured and analysed with the aim of discerning memory accuracy from response bias. For the statistical analyses, a repeated-measures ANOVA, with *valence* (positive, negative), and *arousal* (high, low) as the within-subject factor, and *group* (experimental, control) as the between-subjects factor was calculated. Furthermore, post hoc comparisons (Bonferroni test) were performed to explore plausible differences between conditions. Lastly, *t-*test comparisons were conducted to explore the differences between the two groups in the variables studied for neutral pictures.

All statistical analyses were carried out using SPSS IBM Statistics (Version 28.0).

## Results

### Effect of working memory overload on emotional processing

#### Self-ratings

Regarding the hedonic valence ratings, the repeated-measures ANOVAs revealed a main effect of *valence* (η^2^ =.89; see Table 4). Specifically, post hoc comparisons showed that stimuli with positive valence (*M* = 7.30, *SD* = 1.09) were rated as more pleasant in comparison with pictures with negative valence (*M* = 2.89, *SD* = 1.19, corrected *p* values <.001). Likewise, the analyses also showed a main effect of *arousal* for subjective hedonic valence (η^2^ =.61; see Table 4), with the high arousing stimuli (*M =* 4.09, *SD* = 1.33) being rated as less pleasant compared with the low arousing ones (*M =* 6.09, *SD* =.91, corrected *p* values <.001). Lastly, the analyses revealed no main effect of *group* (see Table 4) for valence ratings (*M* = 5.03, *SD* =.74 for the control group and *M* = 5.15, *SD* =.85 for the experimental group). Regarding interactions, a statistical significance was only observed in the Valence × Group interaction for subjective valence (η^2^ =.086). Post hoc comparisons revealed that the experimental group rated negative stimuli as less unpleasant (*M* = 3.19, *SD* = 1.24) compared with the control group (*M =* 2.58, *SD* = 1.07, corrected *p =*.031; see Table 3).

**Table 3:** Means (*M*), standard deviations (*SDs*) and confidence intervals (CI) for SAM scale

		Control			Experimental		
		95% CI			95% CI		
		*M *(*SD*)	Lower	Upper	*M *(*SD*)	Lower	Upper
ValenceSAM	P-H	6.61 (1.68)	6.00	7.22	6.03 (1.99)	5.40	6.66
	P-L	8.33 (1.04)	8.00	8.68	8.21 (1.04)	7.85	8.56
	N-H	1.72 (1.58)	1.20	2.24	2.00 (1.56)	1.46	2.54
	N-L	3.44 (1.36)	2.94	3.95	4.38 (1.69)	3.86	4.91
	Neut	5.08 (1.48)	4.58	5.58	5.32 (1.89)	4.67	5.98
ArousalSAM	P-H	5.83 (1.86)	5.14	6.53	5.06 (2.29)	4.34	5.77
	P-L	3.36 (2.42)	2.60	4.12	2.74 (2.12)	1.96	3.52
	N-H	6.86 (2.18)	6.16	7.57	6.00 (2.06)	5.27	6.73
	N-L	4.56 (1.70)	3.94	5.18	3.65 (2.03)	3.01	4.29
	Neut	4.47 (1.86)	3.84	5.10	3.74 (2.04)	3.03	4.45

With regard to subjective arousal ratings, the analyses showed a main effect of *valence* (η^2^ =.29; see Table 4). Specifically, post hoc comparisons showed that stimuli with positive valence (*M =* 4.25, *SD* = 1.72) were rated as less arousing in comparison with pictures with negative valence (*M =* 5.27, *SD* = 1.60, corrected *p* values <.001). In the same vein, the analyses revealed a main effect of *arousal* for arousal ratings (η^2^ =.57; see Table 4), with the high arousing stimuli (*M =* 5.95, *SD* = 1.85) being rated as more arousing compared with the low arousing ones (*M =* 3.58, *SD* = 1.72, corrected *p* <.001). Additionally, the analyses revealed a main effect of *group* (see Table 4) for arousal ratings, with the overall experimental group rating stimuli as less arousing (*M =* 4.36, *SD* = 1.52) than the control group (*M =* 5.15, *SD* =1.30, corrected *p* =.022). No significant Valence × Arousal × Group interaction was found*.*

**Table 4:** ANOVA results for self-ratings

	ValenceSAM			ArousalSAM		
	*F*	η^2^	PO^a^	*F*	η^2^	PO^a^
Valence	538***	.89	1.00	28.4***	.29	.99
Val. × Group	6.42*	.086	.70	0.23	.003	.076
Arousal	105***	.61	1.00	89.9***	.57	1.00
Arousal × Group	2.03	.029	.29	0.01	.00	.05
Val. × Arousal	0.12	.002	.063	0.031	.00	.053
Val. × Arousal × Group	0.12	.002	.063	0.063	.001	.057
Group	0.47	.007	.10	5.51*	.075	.64

*Note.* *p <.05. **p <.01. ***p <.001

OP = observed power; Val. = Valence

^a^Alpha =.05

Lastly, we performed a Student’s *t* test between groups for the neutral block, which yielded no significant differences between the experimental and control group for either self-ratings of hedonic valence or arousal (see Table 3).

#### fNIRS activations

The repeated-measures ANOVAs revealed no significant main effects for *valence*, *arousal*, or *group* for any of the brain areas. Regarding the interactions, only that between *arousal* and *group* in region 46 was statistically significant (η^2^ =.08; see Table 6). In this sense, post hoc comparisons revealed that the experimental group exhibited greater activation to high arousal stimuli (*M* =.810, *SD* =.104) than to low arousal stimuli (*M* =.797, *SD* =.094, corrected *p* =.016) in this region (see Tables 5 and 6), while there was no significant Valence × Group interaction or Valence × Arousal × Group interaction (see Table 6). Furthermore, an independent samples Student’s *t* test was performed for the neutral block, finding no significant differences between the experimental group and the control group in any of the areas (see Table 5).

**Table 5:** Means (*M*), standard deviations (*SDs*) and confidence intervals (CI) for fNIRS activity

	Area 9			Area 46			Area 11			Area 32			Area 47		
	95% CI			95% CI			95% CI			95% CI			95% CI		
	*M(SD)*	Lower	Upper	*M(SD)*	Lower	Upper	*M(SD)*	Lower	Upper	*M(SD)*	Lower	Upper	*M(SD)*	Lower	Upper
**Control**															
P-H	.796 (.136)	.749	.842	.793 (.103)	.750	.835	.883 (.204)	.795	.971	.870 (.218)	.786	.954	.862 (.260)	.762	.961
P-L	.793 (.132)	.748	.837	.795 (.088)	.757	.832	.905 (.200)	.821	.989	.887 (.247)	.797	.976	.880 (.260)	.782	.976
N-H	.778 (.115)	.736	.820	.787 (.089)	.748	.825	.886 (.200)	.800	.972	.856 (.199)	.775	.936	.866 (.263)	.762	.970
N-L	.790 (.128)	.745	.834	.790 (.091)	.752	.828	.893 (.201)	.805	.981	.875 (.226)	.790	.960	.867 (.256)	.769	.965
Neutral	.757 (.104)	.716	.798	.759 (.083)	.726	.792	.845 (.187)	.772	.920	.819 (.206)	.737	.900	.838 (.249)	.740	.937
**Exper.**															
P-H	.792 (.104)	.745	.838	.808 (.119)	.765	.851	.855 (.248)	.767	.943	.805 (.216)	.721	.888	.826 (.255)	.726	.926
P-L	.784 (.094)	.740	.828	.792 (.105)	.755	.830	.843 (.235)	.758	.926	.801 (.213)	.712	.890	.806 (.243)	.709	.903
N-H	.796 (.101)	.754	.837	.812 (.110)	.774	.851	.854 (.243)	.768	.940	.812 (.216)	.732	.893	.846 (.276)	.742	.950
N-L	.786 (.100)	.742	.830	.802 (.106)	.764	.840	.862 (.252)	.774	.950	.805 (.215)	.720	.890	.830 (.252)	.732	.928
Neutral	.760 (.095)	.723	.798	.778 (.099)	.739	.817	.814 (.232)	.722	.906	.768 (.206)	.686	.850	.807 (.255)	.707	.908

**Table 6:** ANOVA results for fNIRS activity

	Area 9			Area 46			Area 11			Area 32			Area 47		
	*F*	η^2^	OP^a^	*F*	η^2^	OP^a^	*F*	η^2^	OP^a^	*F*	η^2^	OP^a^	*F*	η^2^	OP^a^
Valence	0.99	0.02	0.17	0.06	0.00	0.06	0.15	0.00	0.07	0.31	0.01	0.09	1.54	0.03	0.23
Val. × Group	3.34	0.06	0.43	3.30	0.60	0.43	1.36	0.03	0.21	2.36	0.04	0.33	3.20	0.06	0.42
Arousal	0.20	0.00	0.07	1.86	0.04	0.27	0.50	0.01	0.11	0.69	0.01	0.13	0.36	0.01	0.09
Arousal × Group	2.10	0.04	0.30	4.59*	0.08	0.56	1.07	0.02	0.17	2.56	0.05	0.35	3.54	0.06	0.46
Val. × Arousal	0.87	0.02	0.15	0.18	0.00	0.07	0.07	0.00	0.06	0.00	0.00	0.05	0.22	0.00	0.08
Val. × Arousal × Group	1.46	0.03	0.22	0.05	0.00	0.06	2.81	0.05	0.38	0.09	0.00	0.06	0.64	0.01	0.12
Group	0.00	0.00	0.05	0.22	0.00	0.08	0.41	0.01	0.10	1.27	0.02	0.20	0.36	0.01	0.09

*Note.* * *p*<.05. **p <.01. ***p <.001.

OP = observed power; Val. = Valence

^a^Alpha =.05

Finally, a paired *t* test was performed in order to examine the fNIRS activation by Brodmann areas. The *t* test revealed that the mean activation in Area 11 was significantly higher than the average overall activation (*p =*.048, Cohen’s* d* =.21), suggesting this region was that with the highest activation. In contrast, the DLPFC areas showed lower activation compared with the overall average (*p =*.016, Cohen’s* d* =.11 for Area 9; and *p =*.021, Cohen´s* d* =.11 for Area 46).

### Effect of working memory overload on recognition memory

In the following analyses, we work with the raw data, considering both “hits” and “correct rejections” as successes, insofar as in both cases the participant's performance was correct. In this sense, the raw score of successes in each type of image ranges from 1 to 10, with 10 being a perfect performance (see Table 7).

**Table 7:** Means (*M*), standard deviations (*SDs*) and confidence intervals (CI) for recognition memory task

	Control			Experimental		
	95% CI			95% CI		
	*M *(*SD*)	Lower	Upper	*M* (*SD*)	Lower	Upper
P-H	9.33 (1.01)	9.00	9.67	8.85 (1.02)	8.51	9.20
P-L	8.17 (1.28)	7.73	8.60	7.26 (1.33)	6.82	7.71
N-H	9.56 (0.97)	9.26	9.86	9.24 (0.82)	8.93	9.54
N-L	9.50 (1.08)	9.13	9.88	8.88 (1.18)	8.50	9.27
Neutral	9.20 (1.29)	8.79	9.66	8.60 (1.28)	8.17	9.06

The ANOVA results showed a main effect of *valence* and *arousal* (η^2^ =.53 and η^2^ =.54, respectively; see Table 8). Specifically, post hoc comparisons revealed that negatively valenced pictures (*M* = 9.29, *SD* =.89) were better recognized than positive ones (*M* = 8.40, *SD* = 1.004, corrected *p* <.001) and, in the same vein, pictures with high arousal (*M* = 9.24, *SD* =.85) were better recognized in comparison with those with low arousal (*M* = 8.45, *SD* =.98, corrected *p* <.001). Likewise, we found a significant main effect of *group* (η^2^ =.11), with the experimental group (*M* = 8.56, *SD* =.84) performing worse on the recognition memory task compared with the control group (*M* = 9.14, *SD* =.84, corrected *p =.*005).

**Table 8:** ANOVA results for the recognition memory task

	*F*	η^2^	OP^a^
Valence	76.4***	.53	1.00
Val. × Group	1.19	.017	.19
Arousal	79.3***	.54	1.00
Arousal × Group	4.09*	.057	.51
Val. × Arousal	34.2***	.33	1.00
Val. × Arousal × Group	0.096	.001	.061
Group	8.3**	.11	.81

*Note.* *p <.05. **p <.01. ***p <.001

OP = observed power; Val. = Valence

^a^Alpha =.05

Regarding interactions, the findings showed a significant Valence × Arousal interaction (η^2^ =.33; see Table 8). In this regard, post hoc comparisons revealed that in positive pictures, recognition was significantly lower when arousal was low (*M* = 7.72, *SD* = 1.31) than when arousal was high (*M* = 9.09, *SD* = 1.02, corrected *p* <.001), whereas recognition was not affected by arousal when negative pictures were presented (corrected *p* =.101). Similarly, when arousal was low, recognition was markedly lower when the valence of pictures was positive (*M* = 7.72, *SD* = 1.31) than when the valence of pictures was negative (*M* = 9.19, *SD* = 1.13, corrected *p* <.001), whereas when arousal was high, hedonic valence was less significant in recognition memory (corrected *p* =.005).

**Fig. 4:**
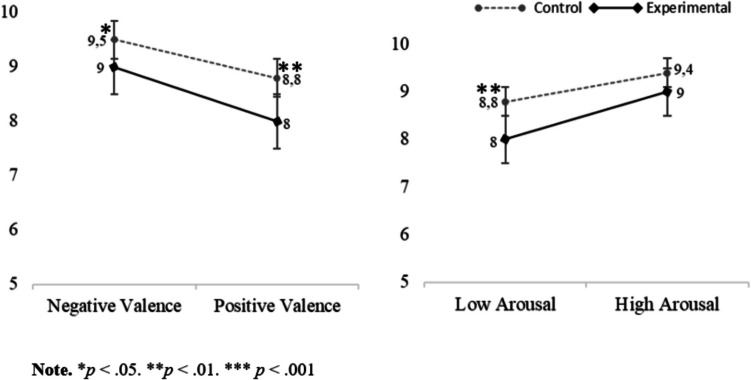
Estimated marginal averages of recognition memory

Additionally, no significant Valence × Group interaction was found. By contrast, there was a statistically significant Arousal × Group interaction (η^2^ =.057). In particular, our post hoc analysis revealed that even if the control group performed better than the experimental group in all conditions, the worsening in the recognition of pictures with low arousal (*M* = 8.83, *SD* =.98) in contrast to those with high arousal (*M* = 9.44, *SD* =.85) was more pronounced and statistically significant only in the control group (corrected *p* =.002; see Fig. 4).

Finally, no overall significant Valence × Arousal × Group interaction was found (see Tables 7 and 8).

**Fig. 5:**
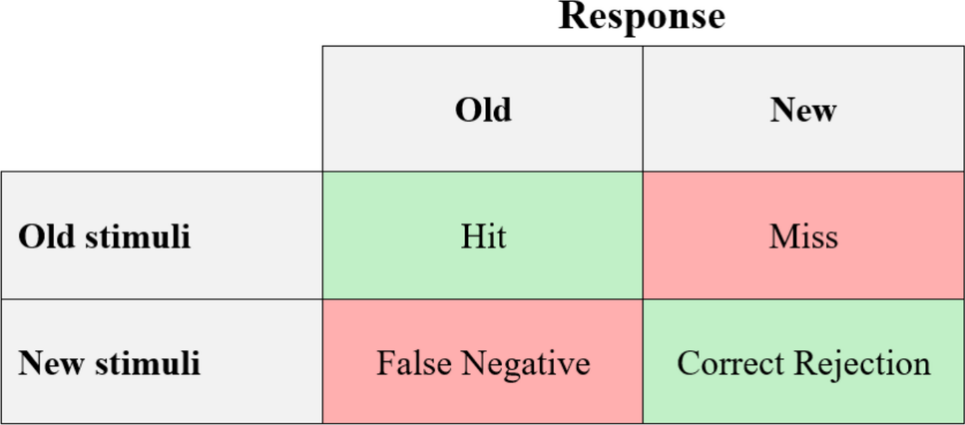
Signal detection theory responses

Additionally, to be able to more comprehensively interpret these results, we estimated the mean response proportions according to the signal detection model (see Fig. 5), through which *d′* and* c* (criterion) were calculated (see Table 9).

**Table 9:** Mean response proportions and summary statistics for each stimulus type

	Control						Experimental				
		P-H	P-L	N-H	N-L	Neutral	P-H	P-L	N-H	N-L	Neutral
Hit		.961	.889	.978	.978	.889	.874	.686	.937	.794	.720
Miss		.039	.111	.022	.022	.111	.126	.314	.063	.206	.280
FA		.106	.250	.061	.061	.044	.103	.234	.097	.046	.011
CR		.894	.750	.939	.939	.956	.897	.766	.903	.954	.989
*d′*		.309	.359	.333	.425	.184	−.327	−.380	−.353	−.450	−.195
*c*		−.153	−.222	−.054	−.278	−.211	.162	.235	.057	.295	.224

*Note.* FA = false alarm; CR = correct rejection

Regarding *d′,* the repeated-measures ANOVAs only revealed a main effect of *group* (η^2^ =.115). Specifically, the control group had a higher *d′* (*M* =.357, *SD* = 1.032) in comparison with the experimental one (*M* = −.377, *SD* = 1.032, corrected *p* =.004), indicating greater accuracy in recognition. The analyses yielded no other main effects or interactions. Additionally, an independent samples Student's *t*-test was performed for the neutral stimuli, finding no significant differences between the experimental group and the control group.

Turning to criterion *c*, the ANOVA showed a main effect of *group* (η^2^ =.124). Post hoc comparisons showed the control group had a lower *c (M* = −.177, *SD* =.492) compared with the experimental group (*M* =.187, *SD* =.490, corrected *p* =.003), suggesting that the control group had a liberal response criterion in comparison with the experimental group, which had a conservative response criterion. In terms of interactions, only the Group × Arousal interaction was statistically significant (η^2^ =.068). Specifically, post hoc analysis showed that, in low arousal stimuli, criterion *c* was significantly lower in the control group *(M* = −.250, *SD* =.582) than it was in the experimental group (*M* =.265, *SD* =.577, corrected *p* <.001), whereas the criterion was not affected by the *group* condition in high arousal stimuli (corrected *p* =.112). Lastly, we performed a Student’s *t* test between groups for the neutral block, which yielded a significantly higher score for the experimental group (*p =*.015, Cohen’s* d* =.73).

## Discussion

The main objective of this research was to study the effect of working memory overload on both emotional processing—evaluated using self-ratings and fNIRS activations—and recognition memory. In order to overload working memory, a concurrent mathematical calculation update task was administered during the presentation of emotional pictures from the IAPS (a classical paradigm used to induce emotions). Likewise, we intended to investigate the effect of hedonic valence and arousal in emotional processing and recognition memory. Additionally, we sought to investigate the role of the DLPFC in working memory and emotional processing, as well as the interaction between the two processes.

Regarding the effect of working memory overload on emotional processing, our findings revealed that the use of a concurrent working memory task during the emotion induction procedure produced changes in the emotional response, both at subjective and brain levels. With regard to self-ratings, the experimental group rated negative pictures as less unpleasant in comparison with the control group. This result goes partially in line with several studies (Kellermann et al., 2012; Kron et al., 2010; Van Dillen et al., 2009) that found the reported feelings decreased when the emotion was induced while a concurrent task was presented. Nevertheless, in contrast to our expectations and some prior results (Kron et al., 2010), we did not find that working memory overload influences the subjective evaluation of emotional arousal. Taken together, these results could suggest that overloading working memory specifically influences the hedonic valence of the pictures, making that negative stimuli are assessed as less negative, but it does not influence on the fact that such stimuli are less emotionally intense. This finding is interesting because it might suggest that the cognitive effort required to maintain information in the short term may act, in some way, as an emotional regulation tool (Mikels & Reuter-Lorenz, 2019). Also, the decrease in the subjective emotional response due to the interference generated by the working memory task is consistent with the idea of the convergence of the neurophysiological correlates of both processes (Gordillo-Ballen, 2014; Jones & Graff-Radford, 2021). In this line, a study shows that the activation of the DLPFC—an area related to working memory—using transcranial direct current stimulation (tDCS) reduced the perceived degree of hedonic valence for negative stimuli (Peña-Gómez et al., 2011), suggesting that the increased activity in this region could bias processing resources towards more cognitive aspects of the stimuli, which may result in the negative stimuli being evaluated as more neutral. This finding goes in line with the results obtained in our study, since we also observed a higher activation specifically in the DLPFC in the experimental group. Surprisingly, fNIRS results did not go in line with the self-ratings, since it was found that the experimental group exhibited greater activation in region 46 of the DLPFC to high arousal stimuli, but not to those with negative valence, contrary to our expectations. Therefore, the fNIRS results showed that this area was sensitized to arousing stimuli when working memory was overloaded, which might demonstrate an interaction between emotional processing and working memory in this region of the DLPFC (Balconi, 2013; Dixon et al., 2017; Funahashi, 2006; Glotzbach et al., 2011; Nejati et al., 2022).

Regarding the outcomes in the recognition memory task, the main effects of valence and arousal confirm that negatively valenced stimuli and those with high arousal were better recognized, in line with our hypotheses. The explanation of these results might lie in the influence of the negative content and arousing stimuli on attention and memory, being interpreted as an adaptive phenomenon in evolutionary terms. In general terms, negative stimuli often carry greater significance for survival because they can signal potential threats or dangers (Lang & Bradley, 2010). Similarly, high arousal may indicate a situation that requires heightened attention and retention, as it may be relevant to an individual's well-being. Therefore, the enhanced recognition observed in response to unpleasant and arousing stimuli may reflect an adaptive mechanism that prioritizes the encoding and retention of crucial information for survival (Gordillo-León et al., 2010; Lazarus, 2021).

Meanwhile, in relation to the effect of working memory overload on recognition memory, the overload task administered to the experimental group generated interference in the recognition memory task. This finding is supported by the higher recognition accuracy of the control group, as revealed by *d′.* However, the results suggest that this interference was less pronounced when identifying high arousal pictures. Specifically, the experimental group showed relatively better recognition of these stimuli, while the improved performance of the control group for them was much less pronounced. In terms of response bias, the experimental group adopted a conservative response criterion, while the control group maintained a liberal criterion. This finding aligns with prior literature (Wylie et al., 2021), as the fatigue induced by working memory overload led to a more conservative response bias, reducing the tendency for false alarms but increasing misses in recognition. These results raise questions about how this effect might influence applied contexts, such as decision-making in high cognitive load environments, where a more conservative criterion could reduce critical errors but also increase omissions.

Furthermore, a significant interaction was observed between valence and arousal in the recognition memory task, showing that the recognition of the positive pictures was weakest, specifically when the arousal was low. This finding is consistent with that reported by Mickley Steinmetz et al. (2010), who found that, at the neurophysiological level, arousal had different effects on the emotional memory network depending on the valence of the stimuli. Specifically, they observed that increased arousal was linked to a greater connection between the hippocampus and cortical areas in the case of images with positive valence, and with a decrease in such connectivity when the images were of negative valence (Mickley Steinmetz et al., 2010).

These results highlight the need to consider valence when exploring the relationship between emotion, memory and neural connectivity inasmuch as understanding this interaction could have applications in the treatment of disorders related to emotional regulation, such as anxiety disorders, depression, or posttraumatic stress disorder (Fitzgerald et al., 2018; LeMoult & Gotlib, 2019; Steward et al., 2022). Surprisingly, this finding runs counter to the premises of the emotional enhancement of memory, which state that emotional stimuli, whether positive or negative, are better remembered than neutral ones (Laney et al., 2004). Finally, following the previous literature (Miendlarzewska et al., 2013), we expected the emotional enhancement of memory to decrease in the experimental group due to the working memory overload. However, a worsening of recognition memory performance in the experimental group due to the working memory overload was not supported for only high arousing items, suggesting that an increased level of arousal compensated for the potential decrease in recognition memory stemming from working memory overload.

This research has several limitations. Firstly, the sample size used in this study was relatively small, in addition to being composed of participants of a similar age range, socio-educational status, and geographic location, which could limit the generalizability of the results to a wider population. Furthermore, the working memory overload may have been influenced by factors such as fatigue (despite block randomization), individual variability, cognitive ability and the type of task itself. For instance, individual variability could have contributed through differences in participants’ baseline working memory capacity, which may affect their ability to process and retain information under conditions of high cognitive demand (Baddeley, 2003). Similarly, cognitive ability may moderate the extent to which participants can deploy compensatory strategies when encountering complex or unfamiliar tasks (Carpenter et al., 2013). Additionally, the type of task itself, being an updating one with a mental arithmetic component, could impose a higher cognitive load on participants less familiar or confident with such operations, amplifying the overload (Ashcraft & Krause, 2007).

Additionally, another limitation is related to the pictures used, which were classified according to the normative values of hedonic valence and arousal (dimensional model of emotions), but we did not consider the specific content of the pictures, which may imply a limitation in the interpretation of the emotional responses. Therefore, it could be interesting to conduct future studies not only based on the dimensional model of emotions, but also on the discrete model, considering the specific categories of the pictures. The dimensional model, which organizes emotions along continuous dimensions such as valence and arousal, has provided valuable insights into the general affective states that influence cognitive processes (Russell, 1980). By contrast, the discrete model of emotions, which classifies emotions into discrete categories, such as happiness, sadness, anger, fear, and disgust, could offer a more nuanced understanding of how specific emotions interact with cognitive functions like working memory (Ekman, 1992). For example, while positive emotions (e.g., happiness) may enhance cognitive flexibility and task performance, negative emotions (e.g., anger or fear) may impede cognitive processes due to their tendency to narrow attention (Isen, 2001). Investigating these discrete emotions could shed light on whether specific emotional states have differential impacts on working memory overload, and how these effects may vary depending on the emotional context presented in the task.

Additionally, considering the interaction between emotional valence and arousal within discrete emotions could provide further insights into their potential moderating effects on cognitive performance. Future studies could employ specific emotional categories within the experimental design to test these hypotheses more rigorously, using both behavioural and neurophysiological measures. Furthermore, it is worth mentioning that, in the design of this research, the hedonic valence was controlled for in the selection of the positive pictures but not in the selection of the negative ones. One of the reasons for this is that it was difficult to obtain negative pictures from the IAPS that were evaluated as very arousing and that were not, in turn, less negative (see Bradley et al., 2001). Generally, the negative pictures rated as low arousing were evaluated as less negative in comparison with the negative picture evaluated as arousing.

Another substantive limitation of this study is the relative novelty of the use of fNIRS as a neuroimaging tool. Given that this tool is an emerging technology in the field of neuroscience, the number of previous studies investigating its application in this context is limited. Therefore, the existing literature may be insufficient to provide a solid frame of reference. While we have attempted to address this limitation through careful design and thorough analysis, it is important to consider that interpretation of the results could be influenced by the lack of comparable research and the constant evolution of fNIRS technology.

Despite the aforementioned limitations, the practical implications of the findings of this study can be applied to several fields. The results of this research suggest that information retention is enhanced with negative and arousing stimuli, which could be especially important in educational settings and learning strategies (McGaugh, 2018; Wang, 2015). Furthermore, understanding how working memory overload affects emotional response may be of interest in the context of mental health and psychotherapy. In this vein, the present line of research could contribute to the design and development of therapeutic interventions focused on emotion regulation and cognitive load management to improve an individual's well-being.

As regards future research, this study opens up several interesting research lines. For example, with the aim of increasing understanding of the relationship between DLPFC, emotions and cognition, future studies could test the underlying neurophysiological correlates using various neuroimaging techniques. Further research using different experimental paradigms and larger and more diverse samples would also be useful in order to replicate the results in different contexts and populations and, therefore, generalize the findings.

## Data Availability

Data will be available on request.

## Footnotes

1 The IAPS numbers used in this experiment were as follows: P-H (4670, 8170, 8186, 8490, 4660, 8501, 8370, 8492, 4290, 8185); P-L (5779, 5760, 5030, 5020, 2530, 2501, 2360, 1670, 1620, 1441); N-H (6230, 3530, 3131, 3130, 3102, 3080, 3068, 3064, 3010, 3001); N-L (5130, 2750, 9000, 9331, 9110, 9090, 7078, 2722, 2590, 2039); neutral (2410, 2396, 2026, 1505, 5120, 5535, 5534, 7491, 7037, 7012).

2 We performed *t*-test analyses to verify that the arousal level was equivalent, regardless of the valence of the stimuli. In this respect, analyses of the comparison between High Valence/High Arousal vs. Low Valence/Low Arousal stimuli showed that those stimuli differed in their terms of valence (*p* <.001) but not in their terms of arousal (*p* >.05). Similarly, the *t* test showed that High Valence/Low Arousal differed in terms of valence (*p* <.001) but not in terms of arousal compared to Low Valence/Low Arousal stimuli. On the other hand, we controlled for the hedonic valence for the positive pictures, but not for the negative ones. Specifically, analyses revealed that the stimuli with High Valence/Low Arousal differed in terms of arousal (*p* <.001) from the High Valence/High Arousal stimuli but not in valence (*p* >.05). However, due to their specific characteristics, we did not control for the effect of valence for the negative pictures. Specifically, we found significant differences in valence and arousal between Low Valence/Low Arousal vs. Low Valence/High Arousal stimuli (*p* values >.001).

3 The following pictures were used for the recognition memory task and were new for the participants: P-H (7405, 4668, 8200, 5621, 8030); P-L (1670, 2035, 5870, 5200, 2388); N-H (3069, 6313, 9940, 3053, 6563); N-L (2399, 6010, 9001, 9280, 2490); neutral (7032, 7001, 7476, 7020, 7000). The following pictures were used for the recognition memory task and were used in the previous task: P-H (4670, 8186, 4660, 8370, 4290); P-L (5779, 5030, 2530, 2360, 1620); N-H (6230, 3131, 3102, 3068, 3010); N-L (5130, 9000, 9110, 7078, 2590); neutral (2410, 2026, 5120, 5534, 7037).
